# Exploring Technical Decision-Making Risks in Construction Megaprojects Using Grounded Theory and System Dynamics

**DOI:** 10.1155/2022/9598781

**Published:** 2022-02-25

**Authors:** Xiaoying Tang, Mengjun Wang, Qian Wang, Jingxiao Zhang, Hujun Li, Juanjuan Tang

**Affiliations:** ^1^Department of Engineering Management, School of Civil Engineering, Central South University, Changsha 410075, China; ^2^Department of the Built Environment, School of Design and Environment, National University of Singapore, Singapore 117566, Singapore; ^3^School of Economics and Management, Chang'an University, Xi'an 710064, China; ^4^Department of Engineering Management, School of Civil Engineering, Henan Polytechnic University, Jiaozuo 454003, China

## Abstract

Technical decision-makings (TDMs) are a vital part of the decision-makings in construction megaprojects, facing high risks brought by technical complexity, dynamic environment, and subject cognition. Identifying technical decision-making risks (TDMRs) and exploring their interactions are important in megaproject management. Due to the high complexity of TDMs in megaprojects, TDMRs are complex and diverse. However, there is a lack of research on exploring the systematic TDMRs in megaprojects. To address this gap in knowledge, this paper aims to better understand the dynamic complexity of TDMRs in megaprojects by identifying the risks and exploring their interactions from a dynamic and systematic perspective. Grounded theory (GT) and system dynamics (SD) were adopted for this research. First, the GT was used to identify TDMRs in megaprojects and create a conceptual model depicting the relationships among TDMRs. Then, an SD model characterizing the causal structure of the TDMRs system in megaprojects is developed in both qualitative and quantitative manners. The developed model involves interrelationships among environmental risks, decision-making process risks, and decision-making execution process risks. After the validation of the model, a model simulation is conducted to predict the dynamic evolution process of the TDMRs. As a result, a multilayer risk list consisting of 42 index layer risk indicators, 13 field layer risk indicators, and 3 standard layer risk indicators is identified. The SD modeling results show that these multilevel TDMRs interact dynamically and have intricate influences on the total risk level of TDMs in megaprojects. The results of this study could be useful for decision-makers to identify and mitigate TDMRs in megaprojects.

## 1. Introduction

Construction megaprojects are characterized by significant technical complexity that requires multitechnology integrations [1–3]. Hence, substantial technical decision-makings (TDMs) are required in megaprojects on almost all management hierarchies [4]. The TDMs refer to the process of identifying and analyzing key problems, as well as developing, selecting, and implementing technical schemes to resolve the problems. Hence, the TDMs include both long-term technology development strategies and short-term technology selections [5, 6]. The outcome of a TDM process is a technical decision-making scheme, consisting of decision objectives, key variables, measures, and criteria [7]. The TDMs must be conducted properly to ensure the successful delivery of megaprojects [2, 7, 8].

Due to the high technical complexity of megaprojects, TDMs in megaprojects also have higher complexity. The complexity of TDMs comprises uncertainties and ambiguities (e.g., dynamic environment, ambiguities of decision-making goals, etc.), as well as the complex interrelationships among influencing factors in TDM issues (e.g., technical complexity relating to the size and multitechnology integration of the project) [9–12]. Thus, TDMs are generally exposed to various risks. Technical decision-making risk (TDMR) is an extension of project risk and decision-making risk [13, 14]. TDMRs are potential hazards existing in the process and outcome of TDM, which negatively affect the TDM quality and project performance. These risks can cause project cost overruns, delays in delivery, and irreversible accidents [15–17]. For instance, in the Busan-Geoje Fixed Link Project, owing to the defective scheme for towing and mooring, the GINA gasket of standard tunnel element E16 was damaged during construction, resulting in a delay of three months and huge financial loss for repairing the GINA gasket [18]. Therefore, effective management of TDMRs in megaprojects plays an important role in successful decision-makings. Further, the TDM in megaprojects is an open environmental system involving multiple subsystems, which has dynamic and complicated relationships among the factors, rather than a series of normative and procedural activities [7, 19]. The dynamic complexity makes TDMRs in megaprojects highly interrelated, and the risks are transmitted between the internal and external environments of the system [20]. Many risk accidents in construction megaprojects occur due to the interactions of multiple risks rather than a single risk [13]. As such, it is imperative to examine the dynamic interactions among TDMRs.

Thus far, a few research efforts have been made to study decision-making risks of megaprojects using both quantitative and qualitative methods, such as optimism-based decision-making risk model for bridge projects [14], decision-making risk mitigation in megaprojects [21, 22], and identifying and assessing specific types of decision-making risks in megaprojects (i.e., design risks [23, 24], technology selection risks [25], social risks [26], and bidding risks [27, 28]). However, the first two kinds of studies were not focused on TDMR, and the last kind of studies was focused on only one specific risk belonging to TDMR (i.e., design risks and technology selection risks). Risks are interrelated and systematic through causal loops in megaprojects [28–30]. Negligence in considering such risk interrelations results in either underestimation or exaggeration of risk effects [12]. Thus, it is crucial to understand how risks are generated and how they transmit through their interactions. It is argued that research is still lacking to explore TDMRs from a systematic and dynamic perspective considering the whole process of decision-making-execution-feedback.

To address the aforementioned research gaps, this paper aims to identify the TDMRs in construction megaprojects and explore their interactions using the mixed method. The grounded theory (GT) is employed to identify all TDMRs in megaprojects, capturing the managers' perception of TDMRs in the practice of TDM in megaprojects. A conceptual framework depicting the relationships among these risks is provided. Then, a system dynamics (SD) model of TDMRs in megaprojects is constructed to explore the casual loops among TDMRs and simulate the interactions among these risks. The contributions of this study lie in two aspects. First, this study investigates TDMRs and their interactions systemically and dynamically for the first time to reveal the dynamic nature of TDMRs, which deepens the understanding of TDM in construction megaprojects and enriches theories of construction decision-making and risk management. Second, the identified TDMRs and the simulation model proposed in this study could be adopted as a tool to evaluate and control TDMRs dynamically. The rest of this paper is structured as follows.  Section 2 introduces the related work, followed by research methodology in Section 3. The results are presented in Section 4, and discussions and implications are illustrated in Section 5. Lastly, Section 6 summarizes and concludes this study.

## 2. Literature Review

### 2.1. Decision-Making Risks in Megaprojects

In recent years, many scholars have indicated the significance of identification and control of the complexity and risks of decision-makings in megaprojects [29, 30]. For example, Shi et al. [7] presented a comprehensive framework of decision-making complexity in megaprojects, which includes six dimensions which are technical, social, financial, legal, organizational, and time. Liu et al. [14] proposed an optimism-based decision-making risk model for bridge projects, where explicit benefits, implicit benefits, construction cost, and operation cost are considered. To mitigate decision-making risks in megaprojects, several researchers proposed risk mitigation strategies via degrading the uncertainty and complexity. Salet et al. [21] put forward three solutions to reduce the complexity and uncertainty of megaprojects to mitigate the decision-making risks, including changing organizational structure, enhancing organizational learning atmosphere, and controlling the number of alternative options for decision-makings.

Furthermore, efforts have been made to identify specific decision-making risks in projects, such as design risks [23, 24], technology selection risks [25, 31, 32], social risks [26], investment risks [33], and bidding risks [27, 28]. Although the aforementioned design risks and technology selection risks belong to TDMRs, each of these existing studies was focused on only one type of TDMRs. Some studies also attempted to assess decision-making risks. For instance, Kurhade and Wankhade [33] proposed a risk assessment framework for decision-making and identified four risk categories for infrastructure investment decision-making, covering political risk, economic risk, social/environmental/cultural risk, and technology risk.

Nevertheless, previous studies on decision-making risks are static without considering the dynamic nature of risks. Attention is lacking to systematically examine TDMRs in construction megaprojects considering the whole process of decision-making. This research gap is addressed in this paper by eliciting the perceptions of managers on what create and drive TDMRs and how they interact with each other by GT.

### 2.2. Risk Assessment Methods in Megaprojects

Risks can be interrelated, especially in megaproject [34]. Megaprojects are characterized by dynamic interactions of multiple subsystems, extreme complexity, and technology challenges [35]. Such dynamic complexity makes the risks in megaproject a dynamic system, where the risks are highly interrelated [36]. To capture the dynamic nature of risks and their complex interactions in megaprojects, various risk analysis methods have been applied, such as interpretative structure models (ISM) [37], complex network (CN) [38, 39], social network analysis (SNA) [40], decision-making trial and evaluation laboratory (DEMATEL) [41], the analytic network process (ANP) [42], Bayesian network, and system dynamics (SD) [36, 43–45].

Among these methods, ISM is a qualitative method aiming to develop the hierarchy structure of the factors with direct and indirect correlation paths, and the developed model is influenced by the number of risk factors [37]. Different numbers of risk factors may result in different hierarchy structure of risk factors. Further, CN, SNA, and DEMATEL aim to analyze risk factors from the network perspective, whereas they failed to evaluate risk state in accordance with the interactions of factors. Meanwhile, ANP and Bayesian network (BBN) can be used to explore the interactions among risks and evaluate the risk state quantitatively, while they require large amounts of data. Wu et al. quantify the risk level of a subway station construction using fuzzy ANP via the synthesis of weight matrices, which requires much more computation for pairwise comparison between risk factors [46, 47]. BBN performs excellently to model complex relationships among risks on the bases of the conditional probabilities of the nodes [48]. However, it can only deal with discrete functions. In recent years, various artificial intelligence (AI) methods, such as machine learning and neural networks, have been utilized to identify, evaluate, and predict potential risks in constructions qualitatively and quantitatively [49]. For example, Yaseen et al. [50] developed an AI model integrating Random Forest classifier and Genetic Algorithm optimization to assess the risk of delay in construction, which indicated a robust and accuracy result for project delay risk prediction. Nevertheless, the abovementioned risk assessment methods analyzed the relationships of risks based on the topology of the network rather than a dynamic and holistic description of the variations of risks. SD developed by Glaser and Strauss [51] is a modeling method dealing with complex causal relationships among components of the system [52]. The foundation of SD is the theory of system thinking, which holds on the view that everything is connected in a dynamic and complex system [53]. SD can not only study the dynamic relationships among risk factors but also simulate risk status during a time period [54]. Hence, SD has been widely used for megaproject risk assessment including modeling of the interrelationships and feedbacks of the risk system. For example, Boateng et al. [55] implemented SD to model the interactions among social, technical, economic, environmental, and political (STEEP) risks considering the complexity and dynamics of megaprojects. Xue et al. [36] proposed a risk coupling model based on SD for risk assessment of High-Speed Rail projects considering the interactions among risks. Wang et al. [56] developed an SD-based safety risk model that covered organizational processes and technical systems and demonstrated the model on an urban metro tunnel project. To identify and control the system risks of automatic metro, Zhao et al. [57] proposed an SD-based model embodying system risks and factors of organizational resource assignment, organizational experience, and avoidance of driver error to reveal the feedback mechanisms of automatic metro. In summary, SD can provide a powerful insight in understanding the complexity and dynamics of construction risk systems [54].

However, there have not been studies exploring the dynamic interactions among TDMRs in construction megaprojects. In this study, SD will be adopted to model the dynamics and interactions among TDMRs.

## 3. Methodology

**Figure 1:**
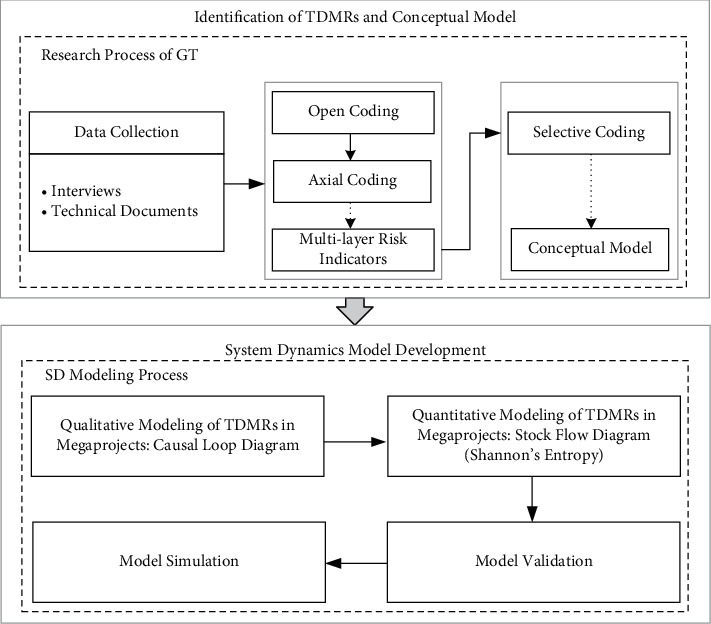
Research framework.

A research framework based on a mixed method integrating GT, SD, and Shannon's entropy was proposed in this study to identify TDMRs and explore their dynamic interactions, as shown in Figure 1. Among the three methods, GT is widely used to identify risks from qualitative data [58], SD is an effective approach for modeling the dynamic relations among risks based on mathematical modeling techniques [59], and Shannon's entropy method is one of the various methods for objective weighting measures. GT, along with qualitative data collection techniques (e.g., case study, interviews, focus groups, etc.) and data analysis techniques (e.g., opening coding, axial coding, and selective coding), can be used to develop SD models based on qualitative data [60]. A mixed method is adopted to draw the advantages and minimize the disadvantages of both qualitative and quantitative methods [61–63]. Several studies have successfully implemented similar mixed methods with GT and SD [53, 64, 65]. For example, in [53], safety archetypes of construction workers were identified by GT and the behavior archetypes of safety involving construction workers were explored with SD.

Therefore, the mixed method was conducted in this study as follows. First, GT was employed to identify TDMRs in megaprojects and develop the conceptual model of these risks, as explained in Section Grounded theory. Then, an SD model of TDMRs in megaprojects was built, which involved qualitative modeling, quantitative modeling, model validation, and model simulation, as discussed in Section System dynamics. Further, the parameters involved in SD equations were determined based on the weights of risk indicators calculated by Shannon's entropy, as introduced in Section Shannon's entropy. Lastly, a simulation was conducted to understand the behavior of the system.

### 3.1. Grounded Theory

This study uses GT to identify TDMRs and build the conceptual model. GT put forward by Forrester [52] as a qualitative research method linking concepts to generate meaningful theories [66], where concepts and their interdependencies are obtained from analyzing qualitative data (e.g., interview transcripts). GT could be applied by three approaches, namely, the Straussian approach [67], the Glaswegian approach [68], and the Constructive approach [69].

Compared to other approaches, the Straussian approach is more prescriptive as it provides systematic procedure for data analysis including open coding, axial coding, and selective coding [70, 71]. On the other hand, the Glaswegian approach and the Constructive approach have no clear guidelines for data analysis. Therefore, the Straussian approach is adopted in this study to identify TDMRs from real megaprojects in a systematic way. Following the Straussian approach, the GT process of this study includes (1) data collection and (2) data analysis consisting of open coding, axial coding, and selective coding, as described in the following [67].

#### 3.1.1. Data Collection

Qualitative data were collected based on a case study so that practical insights could be addressed to enable changes in practice to occur [72]. A case study can include either one single case or multiple cases, and there are different opinions on the required number of cases for understanding a phenomenon [72, 73]. The case study in this research was conducted on three megaprojects in China, namely, the Hong Kong-Zhuhai-Macao Bridge Island Tunnel Project (HZMBIT), the Foshan West Railway Station Comprehensive Transportation Hub Project (FWRSCTH), and the Kunming Comprehensive International Transportation Hub Project (KCITH), to enable generalizations on the TDMRs. These cases were selected because (1) the authors had access to the major stakeholders of these projects, (2) these megaprojects were representative, characterized by multitechnology integrations and high technical complexity, and (3) these projects were under construction or completed less than three years during data collection. The selected cases covered different type of projects (tunnel, railway, and building), and they were all demonstration megaprojects jointly developed by national and local governments. All the cases had multitechnology decision-makings along project lifecycle, which made it possible to collect substantial qualitative data for TDMRs identification.

The case data were collected by semistructured interviews and review of technical documents. Semistructured interviews were conducted with experts from designers, contractors, consultants, and university partners of the three megaprojects. According to Bernard and Bernard [74] and Creswell and Poth [75], interviews with a sample size of 5 to 25 are appropriate for GT studies. In this study, 12 experts were selected for interviews, and the experts had 8 to 27 years of experiences in TDMR management in megaprojects, as shown in Table 1. The selected 12 experts provided meaningful insights that adequately represented the experiences of construction professionals on TDMR management. The interview questions were designed to collect relevant data about TDMRs in megaprojects. The interviewees were asked to elaborate their understandings on TDMRs, provide examples of TDMRs according to their working experience, describe the risk issues, and explain how they deal with risks in the TDM process. For example, the questions asked included (1) what factors drive TDMR events in megaprojects, and what factors contribute to a TDM failure? (2) Do TDMRs interact with each other and how? And (3) what happens if a TDMR event happens in megaprojects? Each of the 12 interviews lasted for 60 to 100 minutes.

In addition to interviews, technical documents of the three megaprojects were also collected to understand the TDMRs in these projects. Due to the large amount of TDMs, the three projects had a large number of technical documents including result-based documents (i.e., construction drawing, technical schemes, and contractual documents) and process-based documents (i.e., safety technical disclosure, environmental checklists and reports, records of technical scheme assessment meeting of the project). A total of 63 technical documents were selected as the raw data including 29 documents from HZMBIT project, 18 documents from FWRSCTH project, and 16 documents from KCITH project.

#### 3.1.2. Three-Level Coding

After data collection, all the collected data were analyzed based on three-level coding, namely, open coding, axial coding, and selective coding. Open coding is an analytic process that identifies the concepts and discovers their properties and dimensions through line-by-line analysis [67]. Hence, in the open coding step, the original data collected from interviews and technical documents were examined and coded to find major TDMRs in megaprojects (i.e., initial codes). Then, axial coding clusters the identified TDMRs into subcategories (i.e., focalized codes) and categories based on their properties and dimensions. Lastly, selective coding was to detect the relationships among different categories [67]. Selective coding is the last step of GT for theory refining and integrating. A conceptual model linking different categories to reveal their relationships was built during this step via reviewing the memos gathered during the analysis and interviews.

### 3.2. System Dynamics

As presented in Section 2.2, SD is used in this study to model the interactions among TDMRs in megaprojects and to reveal how TDMRs interact dynamically and how these interactions contribute to the overall risk. The SD model is developed in the following four steps. In Step 1 (qualitative modeling), based on the identified TDMRs and the conceptual model, system analysis is conducted to draw the system boundary, and the logical structure of the system is defined with a causal loop diagram. Then, Step 2 (quantitative modeling) is performed to formulate the relationships among TDMRs with the stock-flow diagram. Next, Step 3 (model validation) is carried out through structure validation, behavioral validation, and sensitivity validation. Lastly, Step 4 (model simulation) simulates how the system reacts under diverse scenarios.

### 3.3. Shannon's Entropy

Shannon's entropy is capable of measuring the uncertainty of a random process. It is widely used to calculate the weight of each risk indicator based on expert scores [76]. First, the expert scores are normalized using equations (1) and (2) for the-larger-the-better.

Criteria and the-smaller-the-better criteria are, respectively:

(1) $$\begin{array}{c} Y_{ij}=\frac{X_{ij}-\min\left(X_{i}\right)}{\max\left(X_{i}\right)-\min\left(X_{i}\right)}, \end{array}$$

(2) $$\begin{array}{c} Y_{ij}=\frac{\max\left(X_{i}\right)-X_{ij}}{\max\left(X_{i}\right)-\min\left(X_{i}\right)}, \end{array}$$

where *X*_*ij*_ is the score of the i^th^ expert with regard to the *j*^th^ risk indicator (*i* = 1, 2, 3,…,*m*; *j* = 1, 2, 3,…,*n*) and *Y*_*ij*_ is the normalization value of each risk indicator.

It is important to note in this paper that since the experts score based on the importance of each risk indicator, all scores of indicators are processed following the-larger-the-better criteria.

Subsequently, the entropy value *E*_*j*_ of each risk indicator is calculated as follows:

(3) $$\begin{array}{c} E_{j}=-\frac{1}{\ln\;m}\sum_{i=1}^{m}p_{ij}\ln\;p_{ij}, \end{array}$$

where *m* is the number of experts; *p*_*ij*_=(*Y*_*ij*_/∑_*i*=1_^*m*^*Y*_*ij*_). If *p*_*ij*_=0, $\underset{p_{ij}⟶0}{\lim}p_{ij}\ln\;p_{ij}=0$. Then, the weight *W*_j_ of each risk indicator can be calculated as

(4) $$\begin{array}{c} W_{j}=\frac{1-E_{j}}{\sum_{j=1}^{n}\left(1-E_{j}\right)}, \end{array}$$

where *E*_*j*_ is the entropy value of each risk indicator, and *n* is the number of the indicators.

## 4. Results

### 4.1. Identification of TDMRs in Megaprojects

According to the Project Management Institute (2008), a risk is “an uncertain event of condition that, if occurs, has a positive or negative effect on project's objectives.” Following this definition, all the collected data were examined and TDMRs were identified through GT-based data analysis. In the open coding step, by identifying and describing overall constructs relevant to TDMRs based on the collected data, 97 key concepts were extracted through line-by-line and sentence-by-sentence analysis. Then, the 97 key concepts were summarized as 42 initial codes (A1-A42). Next, the axial coding step identified connections between the initial codes and aggregated the initial codes into focalized codes and categories. As a result, the initial codes were grouped into 13 focalized codes (B1–B13) and further into three categories (BB1-BB3).  Table 2 gives some examples of how the collected data were coded in open coding and axial coding.

Table 3 shows the coding results of TDMRs including 42 initial codes, 13 focalized codes, and 3 categories. Due to space limitation, the 97 key concepts are not shown in the table but can be obtained from the corresponding author upon request. According to the coding results, TDMRs in megaprojects are divided into three categories: decision-making process risk, decision-making execution process risk, and environmental risk.

Four layers of risk indicators (shown in Table 4) are established from the coding results including (1) target layer risk indicator (i.e., the total TDMR in a project), (2) standard layer risk indicators (i.e., corresponding to three categories), (3) field layer risk indicators (i.e., corresponding to 13 focalized codes), and (4) index layer risk indicators (i.e., corresponding to 42 initial codes). Risk indicators in each layer are determined by indicators in the lower layers. For example, B1 is determined by A1 to A5, and BB1 is determined by B1 to B5.

Decision-making process risk (BB1) represents risks within the process of identifying and analyzing problem and developing and choosing the technical solution. This process involves decision-makers, information, and procedure, and the outcome is a decision-making scheme. Five field layer risk indicators are related to BB1 including decision-making participants risk (B1), decision-making information risk (B2), procedure risk (B3), decision-making mechanism risk (B4), and decision-making scheme risk (B5).

Decision-making execution process risk (BB2) refers to risks associated with the execution process of the final technical decision-making scheme. Corresponding to the elements and characteristics of decision-making execution, executive, premanagement, in-process management, and technology management are key issues for successful execution of decision-making. Three field layer risk indicators are associated with BB2 including management risk (B6), executive risk (B7), and construction technical risk (B8).

Environmental risk (BB3) describes risks related to the external environment of TDM. The environment contains elements related to society, technology development, economy, and natural and political environment. Five field layer risk indicators are correlated to BB3 including technical environmental risk (B9), economic environmental risk (B10), natural environmental risk (B11), social risk (B12), and political environmental risk (B13).

**Figure 2:**
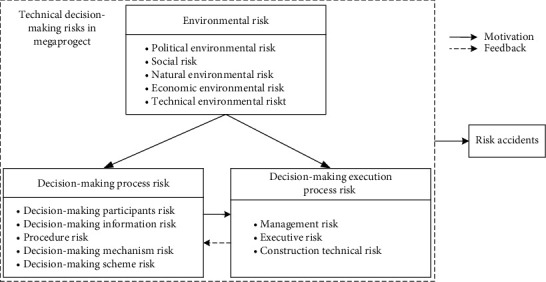
Conceptual model of the relationships among TDMRs in megaprojects.

In selective coding, three categories of risk indicators (BB1-BB3) were linked following a single storyline around which everything else was draped [77]. A conceptual model of their relationships was developed with grounded theory, as shown in Figure 2. The risk lies in the interaction between the subject and the environment [78]. The TDMR accidents in megaprojects occur under the joint influence of the environmental risks, the decision-making process risks, and the decision-making execution process risks. When environmental risks occur, there will be an increased tendency for the risk of the decision-making process and the risk of decision-making execution process. At the same time, the decision-making process risks may transmit to the decision-making execution process risks.

### 4.2. Dynamic Model of TDMRs in Megaprojects

#### 4.2.1. Qualitative Modeling of TDMRs in Megaprojects

**Figure 3:**
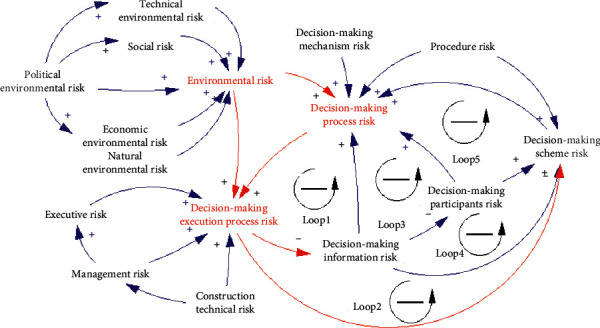
Causal loop diagram of TDMRs in megaprojects.

To qualitatively model TDMRs and understand the feedback loops among TDMRs, a causal loop diagram containing the three categories and 13 focalized codes of TDMRs is depicted, as shown in Figure 3. A causal loop diagram aids in visualizing how TDMRs and variables affect one another by arrows with positive or negative labels (Bala et al., 2017). The diagram is created based on the abovementioned coding results, especially the conceptual model of relationships among TDMRs in megaprojects, as well as the 12 interviews. Furthermore, a group interview was conducted with the abovementioned experts to validate the structure of the diagram. It is noted that the developed causal loop diagram aims to reveal the main causal loops among TDMR. Thus, the index layer risk indicators are not considered in the causal loop diagram since risk indicators in each layer are determined by indicators in the lower layers and the index layer risk indicators are the lowest layer. As presented in Figure 3, TDMRs interact with each other in 3 ways: (1) by the process of decision-making (i.e., the risks lie in decision-making process transmit to the decision-making execution), (2) by the life cycle of the project (i.e., the TDMRs lie in previous construction stage transmit to the next construction stage), and (3) from the external risk to the internal risk (i.e., environmental risks transmit to decision-making process and decision-making execution process). The decision-making process risks may transmit to the decision-making execution process risks. The diagram includes five balancing loops, which interact with each other.

**Loop 1:** Decision-making information risk--(+) Decision-making process risk--(+) Decision-making execution process risk--(-) Decision-making information risk. This is a balancing feedback loop meaning that the increase of decision information risk will stimulate the rise of decision-making process risk, with which decision execution process risk will grow, and then much attention will be paid to decreasing the risk of decision-making information risk. According to Pirzadeh and Lingard [79], technical decision-makings arise as the result of information exchanges between projects actors. Information is essential as an input of the decision-making process [23, 80].

**Loop 2:** Decision-making scheme risk--(+) Decision-making process risk--(+) Decision-making execution process risk--(-) Decision-making scheme risk. This is a balancing feedback loop indicating that the increase of decision-making scheme risk will result in a higher level of decision-making process risk, and then there will be a higher risk during the execution process of decision-making, which will attract more attention and the decision-making scheme will be checked and improved in turn. Decision-making scheme is the outcome of a decision-making process, guiding the execution process [7]. Thus, the decision-making risk could transmit to decision-making execution process imperceptibly until the risk events happen. For example, in the HZMBIT project, the rib rubber mound was initially recommended as the structural design of the artificial island. However, it is found that the scheme may result in quality defects, delay, and pollution in execution process. Therefore, the initial design scheme was replaced by the large diameter deep inserted steel cylinder scheme, which sped up the schedule greatly [81].

**Loop 3:** Decision-making information risk--(+) Decision-making participants risk--(+) Decision-making process risk--(+) Decision-making execution process risk--(−) Decision-making information risk. This is a balancing feedback loop including a part of Loop 1. In addition to the information shown in Loop 1, Loop 3 also illustrates that the decision-making participants are more likely to make wrong decisions with incomplete and inaccurate information, which will result in a higher risk level of the decision-making execution process. Then it will provide feedback to improve the information quality. Research has shown that the knowledge to make a TDM resides in more than one decision-making participant [82]. TDM arises as the result of interactions and information exchanges among decision-making participants [79]. Hence, it is safe to claim that decision-making participants, such as project managers, play a pivotal role in successful TDM [83].

**Loop 4:** Decision-making information risk--(+) Decision-making scheme risk--(+) Decision-making process risk--(+) Decision-making execution process risk--(−) Decision-making information risk. **Loop 5:** Decision-making information risk--(+) Decision-making participants risk--(+) Decision-making scheme risk--(+) Decision-making process risk--(+) Decision-making execution process risk--(−) Decision-making information risk. The balancing Loop 4 and Loop 5 include parts of Loop 1 to Loop 3. Loop 4 and Loop 5 further explain how risks transmit among decision-making information, decision-making scheme, and decision-making participants. The poor quality of decision-making information makes it more difficult for decision-making participants to make decision-making schemes [79]. The timely and effective exchange of information among participants is critical for the development of TDM schemes [84].

#### 4.2.2. Quantitative Modeling of TDMRs in Megaprojects

**Figure 4:**
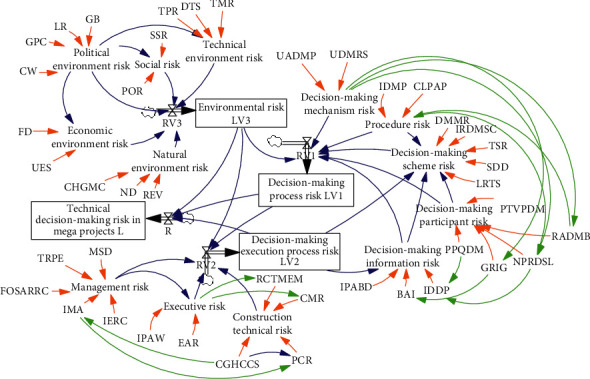
System stock-flow diagram of TDMRs in megaprojects.

To quantitatively model the interactions among TDMRs, it is essential to draw the system stock-flow diagram and build the dynamic equations. Based on the causal loop diagram of TDMRs in megaprojects as well as the characteristics of TDMRs in megaprojects, the system stock-flow diagram with four stock variables, four rate variables, 22 auxiliary variables, and 33 constant variables is built with three subsystems, namely, the decision-making process risk subsystem, environmental risk subsystem, and decision-making execution process risk subsystem. The meanings of SD variables in system stock-flow diagram are shown in Table 5. Arrows connect the four types of variables, indicating either substance or information flow between the two variables. As shown in Figure 4, a set of variables are involved in each subsystem. (1) Subsystem of decision-making process risk: decision-making process risk is quantified with the equations in Table 6. (2) Subsystem of decision-making execution process risk: decision-making execution process risk is a stock variable, which is influenced by the growth rate of decision-making execution with equations in Table 6. (3) Subsystem of environmental risk: environmental risk was determined by the growth rate of environmental risk with corresponding equations in Table 6. In terms of system of technical decision-making risk in megaprojects, technical decision-making risk is a stock variable and the growth rate of the technical decision-making risk in megaprojects as input of stock variable. Further, the growth rate of the technical decision-making risk in megaprojects was influenced by the decision-making process risk, decision-making execution process risk, and environmental risk. The relationships are depicted by equations in Table 6.

The mathematical equations of variables involved in each subsystem are established based on the stock-flow diagram. The coefficients of variables in each equation are established based on the weights of indicators. To determine the weights of indicators, questionnaires were distributed to seven experts engaged in megaproject management and risk management research at universities and practice fields. The experts included two professors engaged in megaproject risk management research at universities, two engineering managers engaged in whole process engineering consulting, two engineering managers from general construction contracting company, and one engineering manager from megaproject owner. They provide evaluations of the importance of each risk indicator in the index layer.

The experts were asked to score based on the controllability, possibility, and degree of loss of the risk. Responses are made based on a five-level Likert scale (1–5), where 1 represents lowest importance and 5 represents highest importance. In general, there are two categories of weighting methods, namely, subjective weighting methods and objective weighting methods [63]. Thereinto, subjective weighting approach is conducted on the basis of decision-maker's experiences and judgment, while the objective weights were calculated via mathematical computation [63]. According to Deng et al. [72], the method with objective weighting is more applicable when it is difficult to obtain the reliable subjective weights. In this paper, all the selected seven experts have much experience in TDMR management, and it is hard to quantify the subjective weights of experts. Therefore, the weights of indicators were obtained via objective weighting method, namely, Shannon's entropy, as illustrated in Section 3.3, ignoring the subjective weights of each expert. The obtained weights of risk indicators are shown in Table 7, and the mathematical equations of variables are presented in Table 6.

### 4.3. Model Validation

Structure validation, behavioral validation, and sensitivity validation are performed to test the structure of the SD model and observe whether the model is consistent with the actual situation [85]. In this study, the structure validation aiming to assess the structural reliability of the model is conducted via structure verification test and dimension consistency test. The variables in the model are extracted from interviews and technical documents, and their relationships are confirmed with a structure verification through interviews with experts. Then, the behavioral validation test is conducted by running the simulation model for the period of one month and comparing the simulation results with the actual field data. The actual field data were collected from the KCITH project, and the comparison shows that the simulation model could produce similar results with the field data.

**Figure 5:**
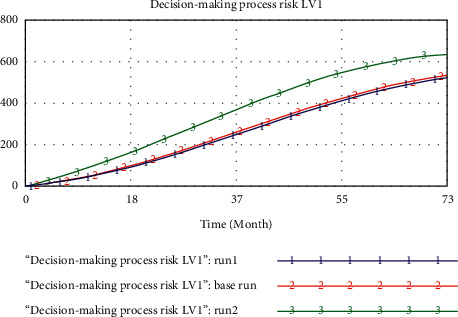
Sensitivity analysis of PTVPDM on the decision-making process risk.

Furthermore, sensitivity validation is used to analyze the effects of the alteration of variables on model simulation results and identify critical TDMRs in megaprojects. Taking the decision-making process risk subsystem as an example, it is found that decision-making process risk is the most sensitive to psychological tendency and value preference of decision-makers (PTVPDM), unreasonable allocation of decision-making power (UADMP), and decision-making method risk (DMMR). The influence of PTVPDM on the decision-making process risk can be estimated by changing the initial values of PTVPDM. When the initial value of PTVPDM varies from 0.4 (run 1) to 4 (base run) and 40 (run 2), the decision-making process risk will increase significantly, as shown in Figure 5.

### 4.4. Model Simulation

#### 4.4.1. Model Parameters

The model simulation of TDMRs was conducted based on case study of the KCITH project, which had an estimated investment of over 900 million USD. The project started in November 2017 and was expected to complete in February 2024. The KCITH project was selected since the TDMs of the project faced a variety of risks such as foundation pit collapse, impact of COVID-19, and policy change, due to the high standard of construction, dynamic external environment, and complex geology and climate conditions. Besides, the construction process involved many high-altitude operations and cross-disciplinary activities. At the time of data collection, the project was under construction and suffered time delays and other various risks in TDMs both internally and externally. Furthermore, the simulation results could help project manager to deal with the TDMRs.

According to the actual construction schedule of the project, the model simulation period was set to 73 months and the step length was one month. To determine the initial values of variables in the model, questionnaire surveys were conducted with seven experts participating in this project, including project managers, chief engineer, university experts, and managers of the project management firm. The questionnaire consisted of an introduction to the research aims and an introduction to the meanings of TDMRs and the scoring rules, which guided the experts to score the risk indicators according to the actual project situation and their experiences. Each risk indicator was scored based on a five-level Likert scale (1–5), where 1 represented very low impact and 5 represented very high impact. Based on the responses, the initial value *v*_*i*_ of risk indicator *i* was calculated as the average score of all experts:

(5) $$\begin{array}{c} v_{i}=\frac{1}{k}\sum_{j=1}^{k}x_{ij}, \end{array}$$

where *x*_*ij*_ was the score given by expert *j* for risk indicator *i* and *k* was the total number of the expert. The obtained initial values of all risk indicators required in the technical decision-making system are shown in Table 8.

#### 4.4.2. Simulation Results

**Figure 6:**
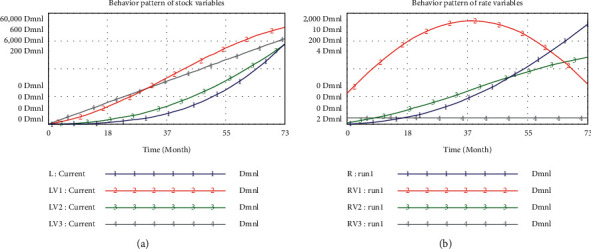
Simulation results of (a) stock variables and (b) rate variables.

Model simulation was conducted to evaluate the evolution of the main stock variables (L, LV1, LV2, and LV3) and rate variables (R, RV1, RV2, and RV3) in the TDMRs system, as shown in Figure 6. According to line 2 shown in Figure 6(a), the decision-making process risk LV1 increases faster at the beginning of the simulation period but then increases with a lower rate. The change of LV1 is consistent with the trend of RV1 (line 2 in Figure 6(b)), which increases at first and decreases after the 37th month. Typically, at the early stage of a megaproject, due to the complexity of the megaproject, decision-making participants lack sufficient cognition of the technical decision-making problem as well as the project information, which contributes to higher decision-making process risk. However, with the accumulation of decision-making execution process risk, some risk accidents may happen, which reveal the problems existing in the decision-making process, and measures (e.g., personnel adjustment and technical scheme adjustment) will be taken to lower the decision-making process risk.

As shown in line 3 in Figure 6(a), the decision-making execution process risk LV2 is very low in the initial several months and then increases with an increasing rate from the 9th month till the end. The corresponding rate variable (RV2) shows a constant increase throughout the period (line 3 in Figure 6(b)). Typically, at the early stage of a megaproject, many decision-making executions process risks are not obvious and the total effect of these risks on the project is weak. Once the technical decision-making scheme is implemented, decision-making execution process risk will keep increasing and the increment rate LV2 also undergoes sustained growth unless the risks are controlled in time.

Furthermore, according to line 4 in Figures 6(a) and 6(b), the environmental risk LV3 keeps increasing at a fixed rate during the whole simulation period, which implies that the environmental risks are constant and not affected by other categories of risks.

Lastly, the *L* (technical decision-making risk in megaprojects) keeps increasing with an increasing rate, as shown in line 1 in Figures 6(a) and 6(b). The result indicates that the total risk will keep extending and lead to risk accidents unless risks are controlled in time. According to Figure 6(a), *L* remains very low before the 18th month and begins to increase faster afterwards, showing a similar trend with LV2. Hence, it can be inferred that LV2 is one of the most significant risk categories. There is also a strong two-way influence between LV1 and LV2. With the implementation of the decision-making scheme and the continuous effect of environmental risks, the decision-making execution process risks gradually accumulate and emerge, easily triggering risk accidents. Once the decision-making execution process is at a high-risk level, many measures will be taken to improve technical decision-making quality, such as to revise the decision-making scheme or to improve the quality of decision-making information.

#### 4.4.3. Scenario Analysis

To provide policy implications for TDMR management in megaprojects, scenario analysis is conducted. For the purpose of clear illustration, only some major variables were selected to examine and describe their effects. First, two scenarios of PTVPDM and ND were selected as examples to conduct single variable analysis, detecting different effects of two variables on the overall TDMR in megaprojects. Second, a multivariate scenario analysis with three variables including PTVPDM, ND, and IPAW is carried out as an example to approximate to the real system.

**Figure 7:**
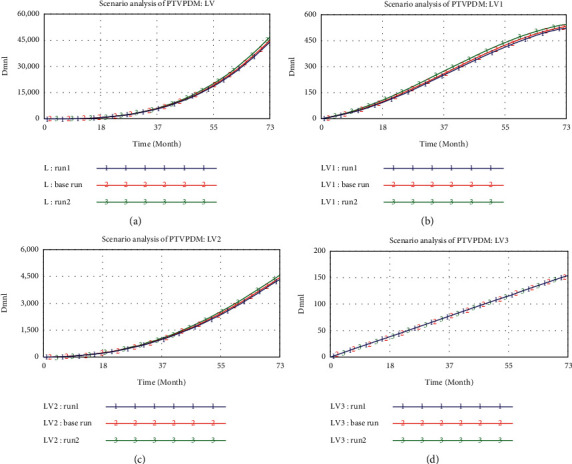
The results of scenario analysis of PTVPDM: (a) curves of L; (b) curves of LV1; (c) curves of LV2; and (d) curves of LV3.

For the single variable analysis of PTVPDM, three different values of PTVPDM are considered including 1 (run1), 4 (base run), and 7 (run2), respectively. As shown in Figures 7(a)–7(c), the increase of PTVPDM can increase the values of *L*, LV1, and LV2, which is in line with the study finding that risk derives from the interaction between people and the environment [86]. The personal characteristics of decision-makers can influence decision-making quality. If the decision-makers have a high tolerance of risk or have insufficient experience, the decision-making process and decision-making execution process may be subject to higher risks. Meanwhile, according to Figure 7(d), the environmental risk (LV3) does not change with different PTVPDM values, which is consistent with the characteristics of environmental risk. The environmental risk subsystem serves as the driver subsystem to the other two subsystems, and itself is hardly influenced by the other two subsystems. Hence, it is suggested that more attention should be paid to the behavioral risk of decision-makers.

**Figure 8:**
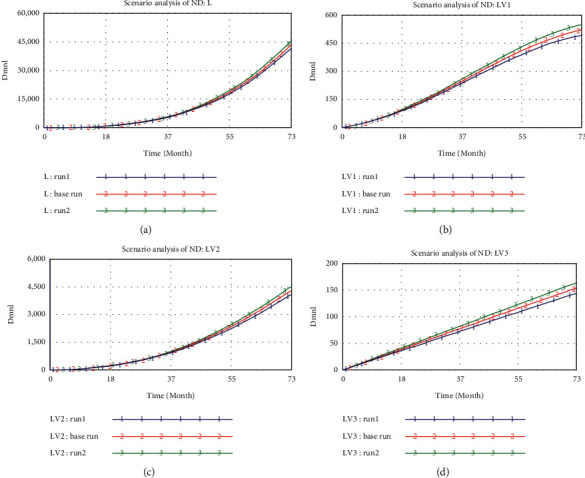
The results of scenario analysis of ND: (a) curves of L; (b) curves of LV1; (c) curves of LV2; and (d) curves of LV3.

For the single variable analysis of ND, three different values of ND, namely, 1 (run 1), 4 (base run), and 7 (run 2), were tested. As shown in Figures 8(a)–8(d), increase of ND will result in increases of all stock variables, which is in line with the influence path of the environment on decision-makings. The increase of ND, which belongs to the environmental risk subsystem, will certainly improve L and LV3, while LV3 will aggravate LV1 and LV2.

**Figure 9:**
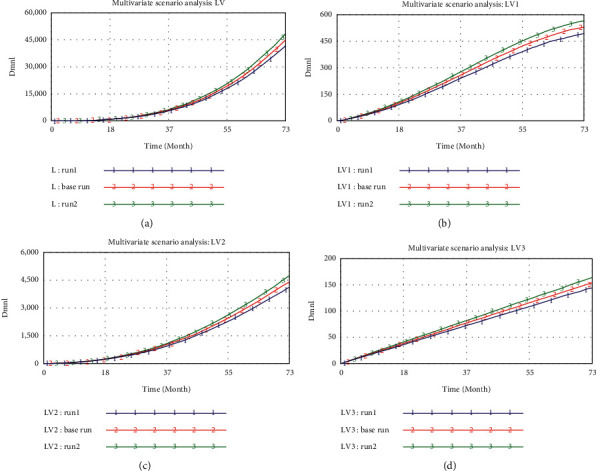
The results of the multivariate scenario analysis: (a) curves of L; (b) curves of LV1; (c) curves of LV2; and (d) curves of LV3.

There are various complicated scenarios in the actual TDMRs system. It is the effect of risk interactions that inflates risk levels, which is the reason why this study explores the TDMRs in megaprojects systematically and dynamically. The multivariate analysis mainly observes the synthesis of PTVPDM, ND, and IPAW by setting the values of them as 1 (run 1), 4 (base run), and 7 (run 2). The results (Figure 9) show that the values of TDMRs are 42119.4 (run 1), 45348.1 (base run), and 48576.8 (run 2), respectively. Compared with the base run, the value of TDMRs decreases by 7.1% (run 1) and increases by 7.1% (run 2), respectively. However, under the scenario analysis of ND, the value of TDMRs decreases by 4.5% (run 1) and increases by 4.5% (run 2) compared with the base run, while under the scenario analysis of PTVPDM, the value of TDMRs decreases by 2.5% (run 1) and increases by 2.5% (run 2) compared with the base run. This implies that the increase of the TDMRs is not relying on the aggregate effects of individual parameters but the synthesis among them. Thus, it is suggested that decision-makers should fully consider how to mitigate the effect of environmental risks in TDM management of megaprojects. The results demonstrate the complex interactions among multilevel TDMRs. A combination of measures considering the comprehensive effects of risks would better control TDMRs in megaprojects.

## 5. Discussion and Implications

The main aim of this research is to identify TDMRs and examine their dynamic interactions. To attain the objective, a hybrid methodology consisting of GT and SD was implemented to explore TDMRs in megaprojects, which combines a qualitative content analysis approach and a quantitative simulation method. GT is used to elicit TDMRs in megaprojects from interviews and technical documents at first. Then an SD model of TDMRs is developed to describe how these TDMRs are interacting with each other, and the dynamic interactions among TDMRs are simulated with different scenarios.

As to the implications, this present research advances our understanding of TDMRs in megaprojects from a systematic and dynamic perspective and can serve as a decision-making management tool for the decision-makers in the following aspects. First, the identified list of TDMRs could be used to evaluate the overall risk level of TDM in megaprojects, which have both theoretical and practical contributions. Second, the SD model representing the interactions among multilevel risks of TDM shows that there are homogeneous and heterogeneous interactions within and among the environmental risk, decision-making process risk, and decision-making execution process risk subsystems. This means that these risk transmissions may aggravate the risk of certain subsystem. Specifically, the results of scenario analysis show that the overall risk level of TDM is inclined to be affected by the synthesis effects of risk interaction rather than the aggregate of individual risks. As such, multilevel measures considering the synthesis effects are more effective to mitigate TDMRs in megaprojects. For example, it is recommended to establish a risk-management-based TDM process, integrating the iterative risk management and TDM process. For each TDM, firstly, identify the risk factors, then, make a decision-making scheme based on the risk status, and evaluate the risk level of the final technical decision-making scheme and modify it dynamically until the risk level of the scheme is acceptable. Besides, a flatter organizational structure for decision-making and multiagent (i.e., the government, the owner, designer, contractor, scientific research institutes, the experts, and equipment suppliers) collaborative decision-making could speed up the information exchange efficiency and reduce risk. In addition, the decision-making information risk, decision-making scheme risk, and decision-making participants risk are three key variables indicated from the five casual loops, which is in line with studies of Sutrisna and Goulding [23] and Eweje et al. [80]. Thus, it is necessary to strictly control the quality of decision-making information and develop a reasonable comparison and selection process of alternative schemes. Finally, the simulation model presented in this paper can be adopted to (1) identify changes of TDMRs over time, (2) evaluate the effects of different risk factors on the total TDMR in megaprojects under different scenarios, and (3) take measures to respond to the project changes brought by TDMRs.

## 6. Conclusions and Limitations

The TDMRs in megaprojects and their interactions are complicated and dynamic, which makes them difficult to control. In the practice of TDMR management in megaprojects, project manager's perceptions of risks may be different from the identified risks in the literature. Therefore, exploring the dynamics of TDMRs fitting the practice of TDM in megaprojects is necessary for both scholars and project managers to gain a better understanding of the complexities of TDMRs in megaprojects. In this study, the TDMRs in megaprojects were identified and a multilayer risk list was determined based on GT. A total of 42 risk factors were identified and classified into 13 subcategories and 3 categories including decision-making process risk, decision-making execution process risk, and environmental risk. An SD model that depicted the dynamic interrelationships among multilevel risks of TDM in megaprojects was built. Rather than exploring single risk's effect, the developed SD model presented the risk-increasing synthesis effects of the interactions among risks.

The results show that the relationships among these TDMRs are complicated. The decision-making process risk and decision-making execution process risk are susceptible to environmental risk, whereas decision-making process risk will transfer to decision-making execution process and decision-making execution process risk may influence the decision-making process in turn. Besides, variables at different levels have varying effects on the total TDMR in megaprojects and the risk level of each subsystem. Among these effects, the synthesis effects of the interactions among risks have a great impact on TDMR in megaprojects. Therefore, it is suggested that a TDM mechanism driven by risk assessment should be established for megaprojects, where only when the risk is in control will the TDM process proceed. Specifically, decision-making execution process risk and decision-making process risk are the two most important risk categories, which need to be paid more attention to.

This study still has several limitations. Firstly, as GT is a qualitative method without quantification and there is a limited amount of original data, the identified TDMRs may be incomplete or inapplicable to other projects. Secondly, the mathematical equations and variable values used in the simulation model are established with from interview data, only considering the objective of each expert, which may not be applicable to other projects. Thirdly, the SD model presented in this paper only depicts the interactions among TDMRs in megaprojects without considering the risk mitigation strategies. Fourth, according to Box et al. [87], at least 50 observations are required to get a useful estimate of the correlation function, while the data used to simulate the model are obtained from the experts rather than practical observation data. Thus, more quantitative indicators and more objective methods (i.e., TOPSIS model for weighting the subjective weights and objective weights) determining the mathematical equations of variables are needed to assess TDMRs in megaprojects. Furthermore, the KCITH project is used for both data collection of GT and model simulation, which may limit the generalizability. Thus, more case studies are necessary to test the applicability and generalization of the presented simulation model [88].

## Figures and Tables

**Table 1:** The personal particulars of interviewees.

Job title	Working experience	Involved project
Project manager	17 years	KCITH
Project manager	17 years	KCITH
Project manager	25 years	HZMBIT
Project manager	22 years	FWRSCTH
Chief engineer	20 years	KCITH
Deputy chief engineer	22 years	HZMBIT
Designer	10 years	FWRSCTH
Designer	8 years	KCITH
Designer	18 years	HZMBIT
Consultant	15 years	HZMBIT
Consultant	13 years	KCITH
Professor	27 years	HZMBIT

**Table 2:** Example of coding process in GT.

Collected data	Open coding		Axial coding	
	Key concepts	Initial codes	Focalized codes	Categories
Decision-maker lacks experience; the requirements of owner are beyond our ability; the chief decision-maker makes decisions based on their own knowledge, experience, and rationale, rather than information from other consulting subjects, which sometimes is impulsive, without enough information. Decision-maker cooperated with the consulting agency in the past; decision-maker usually prefers to choose the scheme he is familiar with rather than the more satisfying one; sometimes, it is hard to control the quality of scheme. Decision-making quality may deviate from the expectations; the consulting agency failed to provide proper advice. The lack of good communication and coordination ability among the decision-making participants leads to instability of the decision-making team. It is hard to coordinate the interests of all parties.	a01 decision-maker lacks ability and professional knowledge	A1 poor professional quality of decision-makers	B1 decision-making participants risk	BB1 decision-making process risk
	a02 the style of decision-maker is different			
	a03 the psychological tendency of decision-makers matters	A2 psychological tendency and value preference of decision-makers		
	a04 the value preference of decision-maker is different			
	a05 decision-making behavior changes	A3 risk of alienation of decision-makers' behavior		
	a06 the advice of experts is not adopted as expected	A4 no prominent role of the decision support layer		
	a07 the consulting agency fails to support the decision-makers			
	a08 unreasonable sharing of risks among project participants in decision-making	A5 game risk of interest groups		

**Table 3:** Coding results of TDMRs including 42 initial codes, 13 focalized codes, and three categories.

Core categories	Categories	Focalized codes	Initial codes
TDMR in construction megaprojects	BB1 decision-making process risk	B1 decision-making participants risk	A1 poor professional quality of decision-makers; A2 psychological tendency and value preference of decision-makers; A3 risk of alienation of decision-makers' behavior; A4 no prominent role of the decision support layer; A5 game risk of interest groups
		B2 decision-making information risk	A6 blocked access to information; A7 improper description of the decision problem; A8 insufficient precision and accuracy of basic data such as survey and design
		B3 procedure risk	A9 incompleteness of decision-making procedures; A10 compliance and legality of project approval procedures
		B4 decision-making mechanism risk	A11 unreasonable allocation of decision-making power; A12 unreasonable decision-making regulation and system
		B5 decision-making scheme risk	A13 decision-making method risk; A14 indicators risk for decision-making scheme comparison; A15 technology selection risk; A16 scheme design defects; A17 the legal risks of the scheme
	BB2 decision-making execution process risk	B6 management risk	A18 timing risk of plan execution; A19 insufficient member ability; A20 fuzzy organizational structure and allocation of rights, responsibilities, and benefits; A21 insufficient emergency response capability; A22 management system defects
		B7 executive risk	A23 insufficient professional ability of workers; A24 the executive's attitude risk
		B8 construction technical risk	A25 changes in geological and hydrological conditions at the construction site; A26 plan change risk; A27 construction and maintenance risks; A28 the risk of construction technology, mechanical equipment, and material
	BB3 environmental risk	B9 technical environmental risk	A29 different technical standard; A30 technology maturity risk; A31 technology policy risk
		B10 economic environmental risk	A32 financing difficulty; A33 unfavorable economic situation
		B11 natural environmental risk	A34 complex hydrological, geological, and meteorological conditions; A35 natural disasters; A36 regional ecosystem vulnerability
		B12 social risk	A37 public opinion risk; A38 social stability risk
		B13 political environmental risk	A39 government behavior; A40 legal risk; A41 government policy changes; A42 coup, war
Total	3	13	42

**Table 4:** TDMRs in megaprojects.

Target layer risk indicator	Standard layer risk indicators	Field layer risk indicators	Index layer risk indicators	Definitions
TDMR in construction megaprojects	BB1 decision-making process risk	B1 decision-making participants risk	A1, A2, A3, A4, A5	Inability of TDM participants to contribute to the decision-making activities and poor collaboration among them
		B2 decision-making information risk	A6, A7, A8	Inappropriate and inaccurate information, lack of documents
		B3 procedure risk	A9, A10	Incompleteness of procedure, lack of standardization, and process records
		B4 decision-making mechanism risk	A11, A12	Lack of rules and regulations
		B5 decision-making scheme risk	A13, A14, A15, A16, A17	Inadequate site investigation, mistakes in the TDM scheme, insufficient comparison, and selection of alternative scheme
	BB2 decision-making execution process risk	B6 management risk	A18, A19, A20, A21, A22	Poor management and supervision in implementation of TDM scheme. Inadequate coordination and collaboration on-site
		B7 executive risk	A23, A24	Inadequate experience and qualification of executive
		B8 construction technical risk	A25, A26, A27, A28	inferior quality and low safety level of the project, due to complex construction
	BB3 environmental risk	B9 technical environmental risk	A29, A30, A31	The uncertainty and immature of new technology. Industry technology is backward
		B10 economic environmental risk	A32, A33	Insufficient supply of capital and required resources and unfavorable macroeconomic situation
		B11 natural environmental risk	A34, A35, A36	Natural disasters, complex geographic and climatic conditions, and high environmental requirements for fragile ecological environment
		B12 social risk	A37, A38	The influence of bad public opinion and the instability of society caused by TDM scheme
		B13 political environmental risk	A39, A40, A41, A42	The uncertainty of the project construction caused by changes in the host country's domestic political situation, legal environment, and political relations with other countries

**Table 5:** Meanings of SD variables.

Variable	Variable type	Meaning
Technical decision-making risk in megaprojects L	Stock	State of technical decision-making risk in megaprojects
BB1 decision-making process risk LV1	Stock	State of decision-making process risk
RV1	Rate variable	The growth rate of the decision-making process risk
B1 decision-making participants risk	Auxiliary variable	
PPQDM	Constant	Poor professional quality of decision-makers
PTVPDM	Constant	Psychological tendency and value preference of decision-makers
RADMB	Auxiliary variable	Risk of alienation of decision-makers' behavior
NPRDSL	Auxiliary variable	No prominent role of the decision support layer
GRIG	Auxiliary variable	Game risk of interest groups
B2 decision-making information risk	Auxiliary variable	
BAI	Auxiliary variable	Blocked access to information
IDDP	Auxiliary variable	Improper description of the decision problem
IPABD	Constant	Insufficient precision and accuracy of basic data such as survey and design
B3 procedure risk	Auxiliary variable	
IDMP	Constant	The incompleteness of decision-making procedures
CLPAP	Constant	Compliance and legality of project approval procedures
B4 decision-making mechanism risk	Auxiliary variable	
UADMP	Constant	Unreasonable allocation of decision-making power
UDMRS	Constant	Unreasonable decision-making regulation and system
B5 decision-making scheme risk	Auxiliary variable	
DMMR	Constant	Decision-making method risk
IRDMSC	Constant	Indicators risk for decision-making scheme comparison
TSR	Constant	Technology selection risk
SDD	Constant	Scheme design defects
LRTS	Constant	The legal risks of the scheme
BB2 decision-making execution process risk LV2	Stock	State of decision-making execution process risk
RV2	Rate variable	The growth rate of decision-making execution process risk
B6 management risk	Auxiliary variable	
TRPE	Constant	Timing risk of plan execution
IMA	Auxiliary variable	Insufficient member ability
FOSBARRC	Constant	Fuzzy organizational structure and allocation of rights, responsibilities, and benefits
IERC	Constant	Insufficient emergency response capability
MSD	Constant	Management system defects
B7 executive risk	Auxiliary variable	
IPAW	Constant	Insufficient professional ability of workers
EAR	Constant	The executive's attitude risk
B8 construction technical risk	Auxiliary variable	
CGHCCS	Constant	Changes in geological and hydrological conditions at the construction site
PCR	Auxiliary variable	Plan change risk
CMR	Auxiliary variable	Construction and maintenance risks
RCTMEM	Auxiliary variable	The risk of construction technology, mechanical equipment, and material
BB3 environmental risk LV3	Stock	State of environmental risk
RV3	Rate variable	The growth rate of environmental risk
B9 technical environmental risk	Auxiliary variable	
DTS	Constant	Different technical standard
TMR	Constant	Technology maturity risk
TPR	Constant	Technology policy risk
B10 economic environmental risk	Auxiliary variable	
FD	Constant	Financing difficulty
UES	Constant	Unfavorable economic situation
B11 natural environmental risk	Auxiliary variable	
CHGMC	Constant	Complex hydrological, geological, and meteorological conditions
ND	Constant	Natural disasters
REV	Constant	Regional ecosystem vulnerability
B12 social risk	Auxiliary variable	
POR	Constant	Public opinion risk
SSR	Constant	Social stability risk
B13 political environmental risk	Auxiliary variable	
GB	Constant	Government behavior
LR	Constant	Legal risk
GPC	Constant	Government policy changes
CW	Constant	Coup, war

**Table 6:** Equations of the variables in the system.

Variable	Equation
BAI	0.213*∗*GRIG, initial value = 1
CMR	0.45*∗*Executive, initial value = 3
Construction technical risk	0.1126*∗*CGHCCS+0.2576*∗*PCR+0.3722*∗*CMR+0.2576*∗*RCTMEM
LV2	INTEG (RV2, initial value), initial value = 0
Decision-making information risk	0.1564*∗*Bai+0.3424*∗*IDDP+0.2564*∗*IPABD-0.2448*∗*LV2
Decision-making mechanism risk	0.5372*∗*UADMP+0.4628*∗*UDMRS
Decision-making participants risk	0.0785*∗*PPQDM+0.5196*∗*PTVPDM+0.0836*∗*RADMB+0.0825*∗*NPRDSL+0.2358*∗*GRIG
LV1	INTEG (RV1, initial value), initial value = 0
Decision-making scheme risk	0.0675*∗*DMMR+0.0675*∗*IRDMSC+0.1028*∗*TSR+0.1319*∗*SDD+0.1352*∗*LRTS+0.113*∗*Decision-making participants risk+0.2057*∗*LV3+0.1146*∗*Decision-making information risk+0.0662*∗*Procedure risk
Economic environmental risk	0.3979*∗*Political environmental risk+0.2198*∗*FD+0.6021*∗*UES
LV3	INTEG (RV3, initial value), initial value = 0
Executive risk	0.2637*∗*IPAW+0.4548*∗*EAR+0.4548*∗*Management risk
GRIG	0.5431*∗*Decision-making mechanism risk, initial = 4
IDDP	0.4876*∗*PPQDM+0.5124*∗*NPRDSL, initial value = 2
IMA	0.4213*∗*CGHCCS, initial value = 3
Management risk	0.2367*∗*FOSARRC+0.1427*∗*MSD+0.3737*∗*TRPE+0.1273*∗*IMA+0.1196*∗*IERC
Natural environmental risk	0.4853*∗*CHGMC+0.1971*∗*ND+0.3176*∗*REV
NPRDSL	0.443*∗*Decision-making mechanism risk+0.322*∗*Procedure risk, initial value = 3
Political environmental risk	0.1416*∗*GB+0.2687*∗*LR+0.1912*∗*GPC+0*∗*CW
PCR	0.456*∗*IMA+0.504*∗*CGHCCS, initial value = 5
Procedure risk	0.2257*∗*IDMP+0.5004*∗*CLPAP+0.2738*∗*RADMB, initial value = 3
R	0.3023*∗*LV3+ 0.3319*∗* LV1+0.3658*∗*LV2
RADMB	0.5431*∗*Decision-making mechanism risk, initial value = 2
RCTMEM	0.4332*∗*Executive risk, initial value = 4
RV1	0.149*∗*LV3+0.1928*∗*Decision-making participants risk+0.1954*∗*Decision-making information risk+0.1053*∗*Procedure risk+0.117*∗*Decision-making mechanism risk+0.2404*∗*Decision-making scheme risk
RV2	0.2148*∗*Management risk+0.1272*∗*Executive risk+0.32*∗*Construction technical risk+0.1611*∗*Environmental risk LV3+0.1769*∗* LV1
RV3	0.1825*∗*Political environmental risk+0.2224*∗*Natural environmental risk+ 0.1661*∗*Economic environmental risk+0.2028*∗*Social risk+ 0.2261*∗*Technical environmental risk
Social risk	0.2454*∗*POR+0.4538*∗*SSR+0.3002*∗*Political environmental risk
L	INTEG (R, initial value), initial value = 0
Technical environmental risk	0.1954*∗*DTS+0.3159*∗*TMR+0.2329*∗*TPR+ 0.2557*∗*Political environmental risk

**Table 7:** Weights of risk indicators.

Target layer risk indicator	Standard layer risk indicators	Field layer risk indicators	Index layer risk indicators	Weight
Technical decision-making risk of megaproject	Decision-making process risk *W*1 = 0.3319	Decision-making participants risk W11 = 0.2266	Poor professional quality of decision-makers	0.0785
			Psychological tendency and value preference of decision-makers	0.5196
			Risk of alienation of decision-makers' behavior	0.0836
			No prominent role of the decision support layer	0.0825
			Game risk of interest groups	0.2358
		Decision-making information risk W12 = 0.2297	Blocked access to information	0.2071
			Improper description of the decision problem	0.5858
			Insufficient precision and accuracy of basic data such as survey and design	0.2071
		Procedure risk W13 = 0.1238	The incompleteness of decision-making procedures	0.3109
			Compliance and legality of project approval procedures	0.6891
		Decision-making mechanism risk W14 = 0.1375	Unreasonable allocation of decision-making power	0.5372
			Unreasonable decision-making regulation and system	0.4628
		Decision-making scheme risk W15 = 0.2825	Decision-making method risk	0.1116
			Indicators risk for decision-making scheme comparison	0.1116
			Technology selection risk	0.3353
			Scheme design defects	0.2180
			The legal risks of the scheme	0.2235
	Decision execution process risk *W*2 = 0.3658	Management risk W21 = 0.3244	Timing risk of plan execution	0.3737
			Insufficient member ability	0.1273
			Fuzzy organizational structure and allocation of rights, responsibilities, and benefits	0.2367
			Insufficient emergency response capability	0.1196
			Management system defects	0.1427
		Executive risk W22 = 0.1921	Insufficient professional ability of workers	0.4837
			The executive's attitude risk	0.5163
		Construction technical risk W23 = 0.4833	Changes in geological and hydrological conditions at the construction site	0.1126
			Plan change risk	0.2576
			Construction and maintenance risks	0.3722
			The risk of construction technology, mechanical equipment, and material	0.2576
	Environmental risk *W*3 = 0.3023	Technical environmental risk W31 = 0.2284	Different technical standard	0.2626
			Technology maturity risk	0.4245
			Technology policy risk	0.3129
		Economic environmental risk W32 = 0.1579	Financing difficulty	0.3188
			Unfavorable economic situation	0.6812
		Natural environmental risk W33 = 0.2246	Complex hydrological, geological, and meteorological conditions	0.4853
			Natural disasters	0.1971
			Regional ecosystem vulnerability	0.3176
		Social risk W34 = 0.2048	Public opinion risk	0.3507
			Social stability risk	0.6493
		Political environmental risk W35 = 0.1843	Government behavior	0.1416
			Legal risk	0.2687
			Government policy changes	0.1912
			Coup, war	0

**Table 8:** Initial risk values of the technical decision-making system.

Variable	Initial value	Variable	Initial value	Variable	Initial value
CGHCCS	2	IDMP	4	PTVPDM	3.13
CHGMC	2	IERC	3	REV	1
CLPAP	3	IPABD	2	SDD	2
CW	0	IPAW	3	SSR	3
DMMR	2	IRDMSC	2	TMR	2
DTS	2	LR	1	TPR	2
EAR	4.23	LRTS	4.3	TRPE	4
FD	1	MSD	2	TSR	3
FOSARRC	2	ND	4	UADMP	3
GB	2	POR	1	UDMRS	3
GPC	5	PPQDM	4.43	UES	4

## Data Availability

The data used to support the findings of this study are available from the corresponding author upon request.
